# Novel technologies: A weapon against tuberculosis

**DOI:** 10.4103/0253-7613.71887

**Published:** 2010-12

**Authors:** B.N. Vedha Hari, Karuna Priya Chitra, Ramadevi Bhimavarapu, Prabhu Karunakaran, N. Muthukrishnan, B. Samyuktha Rani

**Affiliations:** Department of Pharmacy, School of Chemical and Biotechnology, SASTRA University, Thanjavur, Tamil Nadu, India; 1 Department of Pharmacy, S.V. University, Thirupati, Andhra Pradesh, India

**Keywords:** Nanotechnology, polymers, tuberculosis, targeted site

## Abstract

Tuberculosis (TB) is a leading chronic bacterial infection. Despite potentially curative pharmacotherapies being available for over 50 years, the length of the treatment and the pill burden can hamper patient lifestyle. Low compliance and adherence to administration schedules remain the main reasons for therapeutic failure and contribute to the development of multidrug-resistant strains. The design of novel antibiotics attempts to overcome drug resistance, to shorten the treatment course, and to reduce drug interactions. In this framework, nanotechnology appears as one of the promising approaches for the development of more effective medicines. The present review thoroughly overviews the development of novel microparticulate, encapsulation, and various other carrier-based drug delivery systems for incorporating the principal anti-TB agents. Drug delivery systems have been designed that either target the site of TB or reduce the dosing frequency with the aim of improving patient healthcare.

Tuberculosis (TB), a pervasive disease of the respiratory system,[1] is one of the main challenges in public health.[2] The spread of multidrug resistance TB [Multidrug-resistant (MDR)-TB], and the appearance of extensively drug-resistant TB (XDR-TB) pose new challenges for its prevention, treatment, and control. Approximately, two billion people are currently infected with *Mycobacterium tuberculosis* representing about 30% of the global population.[3] The infection is endemic in developing countries, where higher mortality has been reported.[4] It is usually initiated by the entry of the *Mycobacterium* into the respiratory system as aerosol droplets. Bacteria are nonspecifically phagocytosed by alveolar macrophages that process the bacterial antigens and present them to lymphocytes. Then, the number of pathogens increases exponentially by killing host cells and spreading locally to regional lymph nodes in the lungs by lymphatic circulation 3-8 weeks after infection.[5] Later on, spreading of the bacilli from the infected lungs to distant highly irrigated organs [e.g., central nervous system (CNS), spongy bone, liver, kidneys, and genitalia] takes place within 3 months after infection. At this stage, acute TB meningitis or disseminated TB can sometimes result in death. The release of the bacteria to the pleura 3-7 months after infection results in pleurisy. Finally, manifestations (e.g., lesions in bones and joints) can appear. The most effective pharmacotherapy is a multidrug combination of isoniazid (INH), pyrazinamide (PZA), and rifampicin (RMP). During the initial intensive stage (2 months), these three agents are administered together with ethambutol (EMB).[6] The second phase of the treatment for 4 months is exclusively by RMP and INH. These four drugs together with streptomycin (SM) represent the first-line therapy. The prolonged pharmacotherapy and the pill burden can hamper patient lifestyle, and thus, less patient compliance and poor adherence to administration schedules remain the main reasons for therapeutic failure and contribute to the development of MDR strains.[7]

## Symptoms

Latent TB is usually asymptomatic in primary infection but may produce nonspecific symptoms, such as fatigue, weakness, anorexia, weight loss, night sweats, and low-grade fever. In reactivation, symptoms may include a cough that produces mucopurulent sputum, occasional hemoptysis, and chest pain.[8] Early symptoms of active TB include cough, afternoon fever, weight loss, blood stained sputum, and night sweats.

### 

#### Forms of TB

Two forms of TB are latent TB and active TB. In latent TB, the bacteria are dormant in body. This phase can last for a very long time-even decades.[9] It is usually treated by taking one medicine for 9 months. In active TB, the bacteria multiply and spread in the body, thereby causing tissue damage.

#### Multidrug-resistant TB (MDR-TB)

Multidrug-resistant TB is a form of TB in which the bacteria become resistant to at least two first-line drugs INH and RMP. It is primarily the result of patients not taking their full regimen of antibiotics, which allows the bacteria to mutate and develop resistance to the drugs.[10] Some patients with MDR-TB may have contracted it from another person with MDR-TB.

#### Extensively drug-resistant TB (XDR-TB)

XDR TB is a more aggressive form of MDR-TB in which the bacteria are resistant to the first-line drugs[11] namely INH and RMP, any fluoroquinolone, and at least one injectable drug. As with MDR-TB, XDR-TB can be either transmitted or developed. However, the treatment options are fewer and less effective with many unpleasant side effects.

## Drug regimens

### 

#### First-line drugs

The first-line drugs used in treating TB are INH, RMP, PZA, and EMB, and SM [Table 1]. These drugs are administered orally and are shown to have excellent potency against *M. tuberculosis*.[12]

**Table 1:** List of First line anti-tuberculosis drugs

**	*Drug*	*Brand name (company)*	*Dose (mg)*	*Solubility (mg/ml)*	*BCS class*
First line Drugs	Isoniazid	Rimifon^®^ (Roche); Cotinazin^®^ (Pfizer); Ditubin^®^ (Schering); Nydrazid^®^ (Bristol-Myers Squibb).	300	125	III
	Rifampicin	Rifadin^®^ (Sanofi-Aventis);Abrifam^®^ (Abbott); Rifaprodin^®^ (Almirall); Rimactan^®^ (Novartis).	300	0.1	II
	Pyrazinamide	Zinamide^®^ (Merck & Co.); Pezetamid^®^ (Hefa-Frenon); Pyrafat^®^ (Fatol).	500	14	III
	Ethambutol Hydrochloride	Myambutol^®^ (Dura Pharmaceuticals); Etibi, Tibutol.	400	100	III
	Rifabutin	Mycobutin^®^ (Pfizer).	150	0.19	II

N/A: not applicable (administered by i.m. injection), Na: not available.

#### Second-line drugs

The second-line drugs [Table 2] used for the treatment of TB are aminoglycosides such as amikacin (AMK) and kanamycin (KM), polypeptides such as capreomycin, viomycin, enviomycin, fluroquinolones such as ciprofloxacin (CIP), levofloxacin, moxifloxacin (MXF), thioamides such as ethionamide, prothioamide, and cycloserine.[12]

**Table 2:** List of second-line anti-tuberculosis drugs

**	*Drug*	*Brand name (company)*	*Dose (mg)*	*Solubility (mg/ml)*	*BCS class*
Second line drugs	Ethionamide	Trecator^®^ (Wyeth); Nisotin^®^; Trescatyl^®^ (M&B); Aetina^®^; Ethimide^®^; lridocin^®^ (Bayer).	500	0.1	II
	Clarithromycin	Biaxin^®^ (Abbott); Ciathromycin^®^ (Taisho); Klaricid^®^ (Abbott); Naxy^®^ (Sanofi Winthrop); Veciam^®^ (Zambon).	500	0.00033	II
	p-Aminosalicylic acid	PASER^®^ (Jacobus); Rezipas^®^ (Bristol- Myers Squibb).	500	1.7	Na
	Cycloserine	Closina^®^; Farmiserina^®^ (Farmitalia); Micoserina^®^; Oxamycin^®^ (Merck & Co.).	500	100	IV/II
	Amikacin	Sulfate-Amiglyde-V^®^ (Fort Dodge); Amiklin^®^, BB-K8^®^, Biklin^®^ (Bristol-Myers Squibb); Lukadin^®^ (San Carlo); Mikavir^®^ (Salus); Novamin^®^ (Bristol-Myers Squibb).	1000	Na	III
	Kanamycin A	Kantrex^®^ (Bristol-Myers Squibb).	1000	Na	N/A (i.v. or i.m.)
	Capreomycin	Capastat^®^ (Dista), Capastat sulphate^®^ (Eli Lilly).	1000	Soluble in water	N/A (i.v. or i.m)
	Levofloxacin	Cravit^®^ (Daiichi); Levaquin^®^ (Ortho-McNeil); Tavanic^®^ (Aventis); Quixin^®^ (Santen).	500	Sparingly soluble in water	N/A (i.v. or i.m)
	Linezolid	Zyvox^®^, Zyvoxid^®^ (Pfizer).	400	Soluble in water up to 3 mg/ml	Na

N/A: not applicable (administered by i.m. injection), Na: not available.

#### Third-line drugs

The third-line drugs for treating TB include rifabutin, linezolid (LZD), thioidazine, arginine, Vitamin D, macrolides such as clarithromycin (CLR) and thioacetazone (T).[12] The most preferred method of administering the drugs, i.e. through oral route represents many pharmacokinetic problems, such as bioavailability, toxicity, and absorption.[4] The first-line drugs used to treat TB are all orally active. New technologies such as the design of carrier-based drug delivery systems are under investigation for treating TB. To decrease the dose and duration of treatment, biodegradable polymers and liposomes and microsphere carriers were developed. Doses of the drugs are trapped in these forms of carriers and administered either intravenously or subcutaneously.[13] The drugs are gradually released overtime as they diffuse out into the body. The drug diffuses out in concentrations that are large enough to exhibit adequate inhibition of *M. tuberculosis*.

This article reviews the state-of-the-art technologies in the development of nanobased drug delivery systems for encapsulation and to provide target release of anti-TB agents, and discusses the development of a more effective, compliant, and affordable pharmacotherapy for TB.

## Nanotechnologies applied to the treatment of TB

### 

#### Nanosuspensions

Nanosuspensions are submicron colloidal dispersions of pure drugs stabilized with surfactants. Nanonization (reduction of the average size of solid drug particles to the nanoscale generally by top milling or grinding) is an useful methodology to improve the solubility of drugs displaying strong solute-solute interactions and high-melting points, in general, both poor water and lipid solubility. The solid and dense state of the pure drug particles gives a maximal mass per volume ratio, especially critical in systems demanding high drug loadings. An extensive size and size distribution characterization indicate that the nanosuspensions display more homogeneous size distributions. Different solvents are used to solubilize the drug such as dimethyl sulphoxide methanol, ethanol, and ether. DMSO was the most appropriate for nanonization. Spherical particles of various sizes with mean diameters between 400 nm and 3 mm were produced, and sizes were tuned by changing the conditions of the process. These preliminary results would support further investigations to evaluate the potential of this approach as a more convenient TB pharmacotherapy, especially for the localized delivery of anti-TB drugs to the lungs. *In vivo* assays were conducted with nanoformulations containing drug concentrations between 0.16% and 0.18%. Nanocrystalline suspensions of poorly soluble drugs such as riminophenazines and clofazimine[14] are easy to prepare and to lyophilize for extended storage and represent a promising new drug formulation for intravenous therapy of mycobacterial infections.

**Nanoemulsions:** Nanoemulsions are thermodynamically stable oil-in-water (o/w) dispersions displaying drop sizes between 10 and 100 nm.[15] An advantage of these systems is that they are generated spontaneously and can be produced in a large scale without the need of high homogenization energy. In addition, they can be sterilized by filtration. The enhanced uptake of nanoemulsions by cells of the phagocytic system reported elsewhere renders these nanocarriers passive targeting features. In addition, they are taken up by lipoprotein receptors in the liver after oral administration. Resazurin nanoemulsion remains bactericidal for all strains tested. Results reveal that they have a potential role as a novel antimicrobial agent for the treatment of infection due to cystic fibrosis (CF)-related opportunistic pathogens.[16]

#### Niosomes

**Figure 1:**
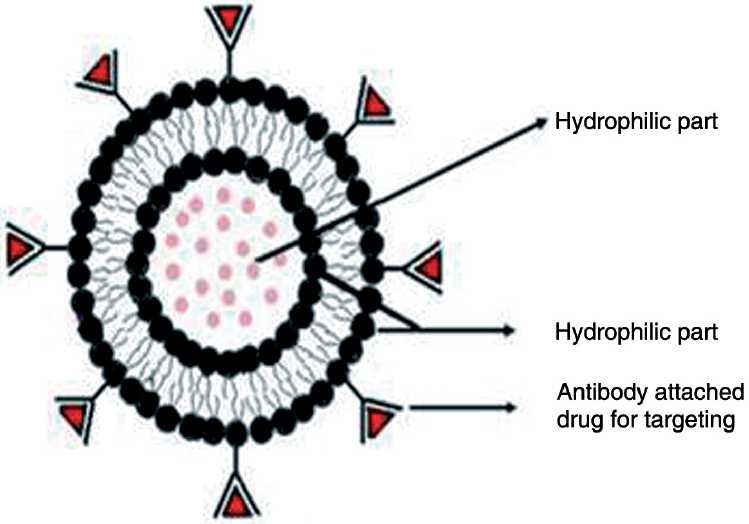
Structure of niosome Source: http://www.pharmainfo.net/files/images/stories/article_images/Niosome%20structure.

Niosomes are biodegradable, biocompatible, and nonimmunogenic in nature and exhibit flexibility in their structured characterization.[17] They are thermodynamically stable liposome-like vesicles produced with the hydration of cholesterol, charge-inducing components such as charged phospholipids (e.g., dicethylphosphate and stearyl amine), and nonionic surfactants (e.g., monoalkyl or dialkyl polyoxyethylene ether). They are associated with advantages such as entrapment of more substances, higher stability, no need of handling or storing in special conditions, and the availability and inexpensiveness of prepared materials. Niosomes can host hydrophilic drugs within the core and lipophilic ones by entrapment in hydrophobic domains as shown in Figure 1. The prepared microsized (8–15 mm) RMP-loaded niosomes contain Span 85 as the surfactant.[18] *In vivo* studies have showed that by adjusting the size of the carrier, up to 65% of the drug can be localized in the lungs. When methotrexate 100 nm C16G3 niosomes containing either 47.5% or 30% cholesterol were administered intravenously or orally, higher levels of the drug were found in the liver, more so for the formulations administered by the intravenous route, with serum levels higher than when the drug was administered in solution.[19] Niosomes have been used for improving the stability of entrapped drug RMP.

#### Polymeric and nonpolymeric nanoparticles

Polymeric nanoparticles (PNPs) have been extensively explored as means for drug solubilization, stabilization, and targeting.[20] The advantageous features such as high stability, high loading capacity of hydrophilic and hydrophobic drugs, and feasibility of administration by different routes have made PNPs one of the most popular approaches for drug encapsulation.[21] Depending on the technology employed for their production, two kinds of systems can be generated, namely nanocapsules and nanospheres. In the former, the drug solubilized in aqueous or oily solvents is surrounded by a polymeric membrane. In contrast, the latter is comprised of solid matrices of variable porosity where the active molecules are homogeneously distributed through the particle and, often, dispersed at the molecular level. A broad spectrum of biomaterials is available for the production of PNP.[22] PNP are removed from the body by opsonization and phagocytosis.[23] To prevent recognition by the host immune system and to prolong circulation times in the blood stream, the modification of the surface with highly hydrophilic chains (e.g., polyethylene glycol) has been pursued, this approach was one of the most extensively investigated with respect to antituberculous drug delivery systems. The encapsulation of RMP, INH, and SM was done within poly(*n*-butylcyanoacrylate) (PBCA) and poly(isobutylcyanoacrylate) (PIBCA) nanoparticles and tested for the accumulation in human blood monocytes *in vitro* toward the development of a drug depot. Encapsulated INH, SM and RMP showed 4-8 and 22-25-fold increase in the intracellular concentration with respect to the extracellular concentration. Du Toit and collaborators developed INH-loaded polymer-based nanosystems by means of a salting-out approach (nanoprecipitation).[24] Two kinds of precursors were used; water- or emulsion-based systems. Nanosystems appeared as spherical nanoparticles with a broad size range (77-414 nm) embedded within a micro- or nanomatrix support. The size of the nanoparticles was adjusted by changing the polymer concentration; a decrease in the concentration resulted in smaller particles. Emulsion-based nanoformulations displayed a more compact architecture. Formation of nanoparticles relied on the interactions of polymer chains within the polymer-droplet interface involving reduction of the interfacial tension and mechanical stabilization in the dispersant electrolyte solution.

#### Polymeric micelles and other self-assembled structures

**Figure 2:**
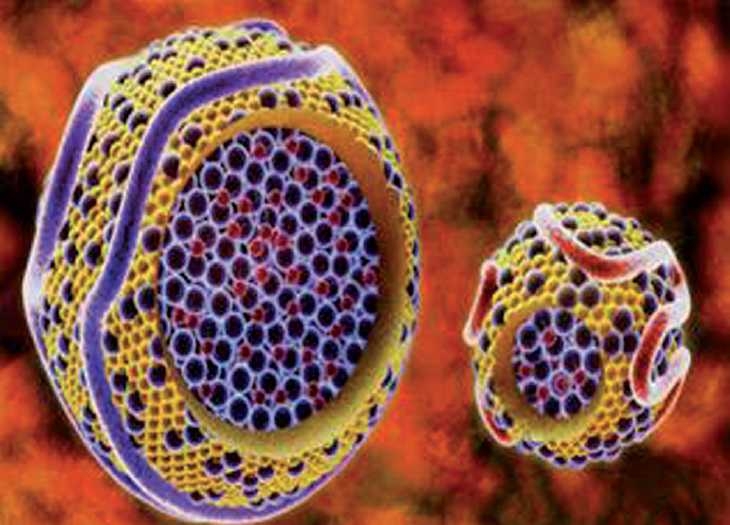
Structure of polymeric micelles Source: http://www.mcgill.ca/files/endocrinology/lipid-particles.jpg

Polymeric micelles are nanocarriers generated by the self-assembly of amphiphilic polymers in water above the critical micellar concentration (CMC).[25] The hydrophilic blocks are exposed to the aqueous medium forming the micellar shell that facilitates the solubilization of the amphiphile in water and stabilizes the aggregate [Figure 2]. In contrast, the hydrophobic blocks form the inner micellar core, a hydrophobic domain that enables the incorporation of poorly water soluble drugs by physical interaction or chemical conjugation leading to higher solubility extents.[26] In addition, sensitive drugs hosted within the core are protected from chemical and biological degradation processes. Polymeric micelles are more stable than conventional micelles (e.g., Tween 80), even at concentrations below the CMC.[27] Commercially available and FDA-approved polyethylene oxide poly(propylene oxide) (PEO-PPO) block copolymers (linear poloxamers and branched poloxamines) are among the most important micelle-forming materials. To sustain the delivery of RIF, drug-loaded stereo complex micelles were produced by the specific assembly of enantiomeric poly(ethylene glycol)-poly(D-lactide) (MPEG-PLLA) and poly(ethylene glycol)-poly(d-lactide) (MPEG-PDLA) block copolymers in a 1:1 ratio of L-poly lactic acid (L-PLA) and D-poly lactic acid (D-PLA) containing block copolymers.[28] An increase in the length of the PLA segment resulted in lower CMC values and larger nanoaggregates. In addition, the drug release time *in vitro* could be controlled by the polymer molecular weight. Wu *et al*. designed PLA-modified chitosan oligomers capable of aggregating in water to form spherical micelles with sizes between 154 and 181 nm. Incorporation of 10% RIF into the nanocarriers led to a slight core expansion to sizes in the 163–210 nm range. *In vitro* release experiments showed a burst effect (35% within 10 h) and a more sustained release until day 5.

#### Dendrimers

**Figure 3:**
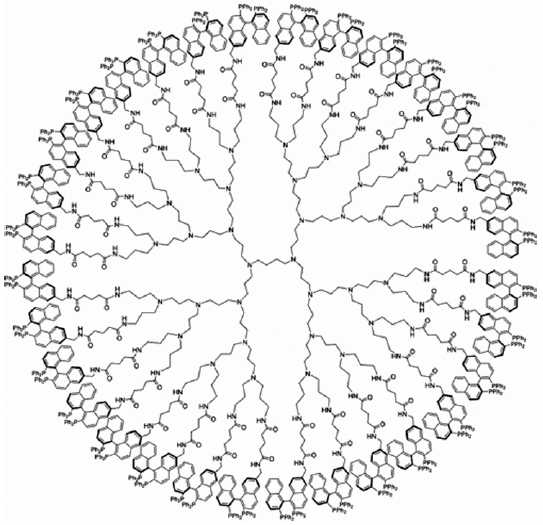
Structure of dendrimer Source: http://www.gade.uni-hd.de/graphics/dendrimers.gif

Dendrimers are macromolecules displaying well-defined, regularly hyperbranched and are three-dimensional structures, relatively low molecular weight polydispersity, and high with adjustable functionality. These functional molecules are feasible for drug encapsulation by virtue of the dendrimeric core and complexation and conjugation on the surface as shown in Figure 3.[29] Due to this unique structure, these molecules represent attractive candidates for the encapsulation and delivery of anti-TB agents for diverse administration routes. Cell compatibility assays have demonstrated the toxicity of the amine-terminated dendrimers. The PEGylation with various systems was found to have increased their drug-loading capacity, reduced their drug release rate, and hemolytic toxicity. The systems were found suitable for prolonged delivery of RMP.[30]

#### Complexation with cyclodextrin (CD)

CD are cyclic oligosaccharides composed of 6 to 12 D-(+)-glucopyranose units linked by α-(1-4) bonds.[31] CD displays a toroid structure that combines a hydrophilic outer surface (due to the presence of hydroxyl moieties) and a hydrophobic inner cavity. This structure enables the partial or complete inclusion of hydrophobic molecules into the cavity; water solubility increases up to several orders of magnitude, as compared to the uncomplexed drug. The aqueous solubility of the drug increased linearly with the concentration of the CD, with the CD/RIF ratio in the complex being 1:1. In addition, the solubilization was pH dependent. CD are associated with advantages such as control of drug delivery as well as site-specific drug delivery, drug safety, drug stability, etc. *For example*, inclusion complex of anti-tubercular RMP with β-cyclodextrin[32] showed that RMP with β-CD and HP-β-CD protects the drug, controls the release rate of the drug, and improves the stability of RMP in fixed dose combinations (FDCs).

#### Liposomes

**Figure 4:**
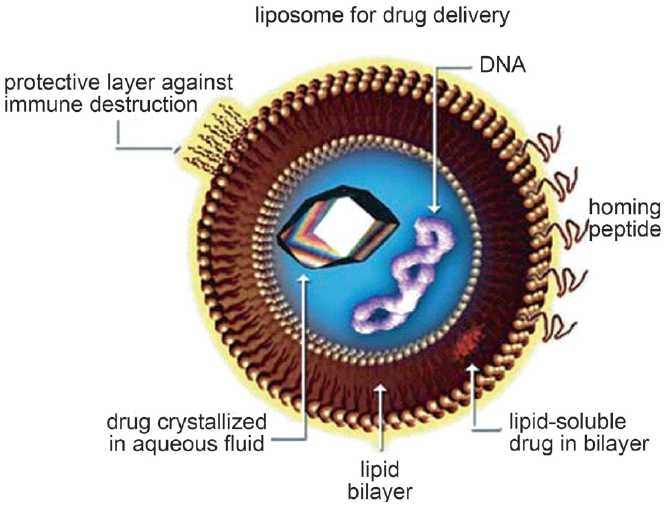
Liposome for drug targeting Source: www.currentprotocols.com/protocol/ps0522

Liposomes are nano- to microsized vesicles comprising a phospholipid bilayer [Figure 4], which surrounds an aqueous core[32] while the core enables the encapsulation of water-soluble drugs, the hydrophobic domain can be exploited to entrap insoluble agents. When administered, these carriers are recognized by phagocytic cells and are rapidly cleared from the blood stream. To prevent elimination and extend circulation times, liposomes are usually PEGylated. In more recent investigations, PYZ[33] and rifabutin-containing liposomes were also produced, stressing the great versatility and potential of these nanocarriers. Reports with INH and rifampin encapsulated in lung-specific stealth liposomes against *Mycobacterium tuberculosis* infection[34] revealed that liposome-encapsulated drugs at and below therapeutic concentrations were more effective than free drugs against TB.

#### Local delivery of anti-TB drugs

Most of the TB manifestations are observed mainly in the respiratory system, local delivery to the lungs by inhalation has emerged as one of the most attractive administration routes to target the TB infection’s cellular reservoir (alveolar macrophages), while reducing systemic adverse effects. Nanotechnology platforms (e.g., PNP and liposomes) previously investigated with the aim of optimizing different technological aspects of anti-TB drugs are currently being explored for the targeted delivery to the lungs.[35] Different industrial techniques, such as spray drying are being employed to produce nanoparticles made up of a variety of natural (e.g., gelatine) and synthetic (e.g., polybutylcyanoacrylate) polymers.[36] Lung lesions containing large numbers of bacteria are poorly vascularized and are fortified with thick fibrous tissue; conventional therapy by the oral and parenteral routes may provide subtherapeutic levels of anti-TB drugs to these highly sequestered organisms. Administering drugs by the pulmonary route to the lungs[37] allows higher drug concentrations in the vicinity of these lesions. Supplementing conventional therapy with inhaled anti-TB therapy may allow therapeutic concentrations of drug to penetrate effectively into lung lesions and treat the resident mycobacteria.

#### Microencapsulation

**Figure 5:**
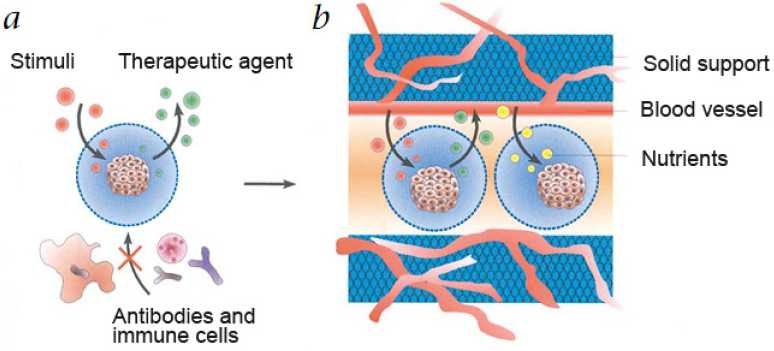
Cel microencapsulation by therapeutic TB agents Source: wiki.epfl.ch/…/encapsulated-cell-technic

Microspheres, on the other hand, are discrete particles. As with liposomes, biological agents can either be encapsulated within the microsphere or attached to the surface. The use of polymers such as lactide-co-glycolide polymers allows significant flexibility with respect to the time at which encapsulated material can be released. Poly(lactide-co-glycolide) microspheres are degraded by hydrolysis only and are not susceptible to enzymatic degradation. The advantage of microspheres would be to extend the time for drug release from days to months with the small microspheres and for a year or more with large microspheres. Microsphere formulations using polymeric excepients of lactide-co-glycolide polymers are biocompatible, the metabolic by-products such as lactic acid and glycolic acid[38] composition is based on the formulation for synthetic resorbable sutures, which degrade by nonenzymatic reaction,[39] which have demonstrated the effectiveness of smaller microspheres for the delivery of rifampin to host macrophages to significantly reduce levels of intracellularly replicating *M. tuberculosis*. The small microspheres were more efficient at delivering effective doses of RMP intracellularly than equivalent doses of free drug.[40,41] A combination of small- and large-microsphere formulations would be ideal for use in TB treatment regimens because a drug could be targeted to host macrophages with the small microspheres and delivered systemically by means of the large microspheres [Figure 5].

## Challenges and Future Prospects

"Age and prior exposure bring no such immunity against TB."[42] Pulmonary TB is the commonest form of TB and alveolar macrophages are the abode of *Mycobacterium tuberculosis* the administration of anti-tubercular drugs via the respiratory route is an exciting possibility. The objective to eliminate the transmission of causative organism is currently out of reach due to difficulty of diagnosis, multidrug resistance, and treatment adherence. The currently available antitubercular drugs should be modified in such a way as to release drugs in a slow and sustained manner, thus it would be possible to reduce the dosing frequency thereby improving compliance. Plain liposomes and other colloidal carriers are largely unsuccessful in drug targeting due to their difficulties in gaining access to targeted tissues, penetrating vascular barriers, and evading phagocytic capture by the reticulo-endothelial system. The advent of the novel drug delivery systems holds the key for the prevention of TB disease.

## Conclusion

Drug-resistant TB plays a major challenge for the effective control of TB. The drugs currently in use were developed 40 years ago, and there is a great need for a new generation of antituberculous drugs. Current research involves testing new or reformulated drugs, combinations of different drugs to shorten therapy, supplementation, and enhancement of existing drugs, development of novel slow-release drug delivery systems that could reduce the frequency and amount of drug necessary during treatment. The goal is to find better and more effective drugs that reduce time of treatment, reduce toxicity associated with drugs and provide backup measures in the case of drug resistance. The race to develop new and more effective TB drugs is on. It is hoped that new more effective drugs will be discovered in the not-too-distant future that could shorten the therapy from 6 months to a few weeks and improve control of this disease worldwide.

## References

[1] Kaufmann SH, McMichael AJ (2005). Annulling a dangerous liaison: Vaccination strategies against AIDS and tuberculosis. Nat Med.

[2] Gaspar MM, Cruz A, Fraga, Castro AG, Cruz MEM, Pedrosa J (2008). Developments on Drug Delivery Systems for the Treatment of Mycobacterial Infections. Curr Top Med Chem.

[3] Tuberculosis. The International Union against Tuberculosis and Lung Disease (The Union).

[4] Du Toit LC, Pillay V, Danckwerts MP (2006). Tuberculosis chemotherapy: Current drug delivery approaches. Resp Res.

[5] Smith I (2003). *Mycobacterium tuberculosis* pathogenesis and molecular determinants of virulence. Clin Microb Rev.

[6] Onyebujoh P, Zumla A, Ribeiro I, Rustomjee R, Mwaba P, Gomes M (2005). Treatment of tuberculosis: Present status and future prospects. Bull. World Health Organ.

[7] Dye C (2006). Global epidemiology of tuberculosis. Lancet.

[8] (2005). Springhouse. Professional guide to disease.

[9] http://www.health.state.mn.us/divs/idepc/diseases/tb/phaseschart.html.

[10] Iseman MD (1993). Treatment of multidrug – resistant tuberculosis. N Engl J Med.

[11] Center for Disease Control (2006). Emergence of *Mycobacterium tuberculosis* with extensive resistance to second-line drug-Worldwide, 2000-2004. MMWR Wkly.

[12] Grange JM, Zumla A (2002). The global emergency of tuberculosis: What is the cause?. The Journal of the Royal Society for the Promotion of Health.

[13] Showalter HD, Denny WA (2008). A roadmap for drug discovery and its translation to small molecule agents in clinical development for tuberculosis treatment. Tuberculosis.

[14] Peters K, Leitzke S, Diederichs JE, Borner K, Hahn H, Muller R H (2000). Preparation of a clofazimine nanosuspension for intravenous use and evaluation of its therapeutic efficacy in murine Mycobacterium avium infection. J Antimicrob Chemother.

[15] Constantinides PP, Chaubal MV, Shorr R (2008). Advances in lipidnanodispersions for parenteral drug delivery and targeting. Adv Drug Del Rev.

[16] Seki J, Sonoke S, Saheki A, Fukui H, Sasaki H, Mayumi T (2004). A nanometer lipid emulsion, lipid nano-sphere (LNS), as a parenteral drug carrier for passive drug targeting. Int J Pharm.

[17] Varaporn Buraphacheep Junyaprasert, Veerawat Teeranachaideekul, Tasaneeya Supaperm (2008). Effect of charged and non-ionic membrane additives on physicochemical properties and stability of niosomes. AAPS Pharm Sci Tech.

[18] Azmin MN, Florence AT, Handjani-Vila RM, Stuart JFB, Vanlerberghe G, Whittaker JS (1985). The effect of non-ionic surfactant vesicle (niosome) entrapment on the absorption and distribution of methotrexate in mice. J Pharm Pharmacol.

[19] Soppimath KS, Aminabhavi TM, Kulkarni AR, Rudzinski WE (2001). Biodegradable polymeric nanoparticles as drug delivery devices. J Control Release.

[20] Delie F, Blanco-Prieto MJ (2005). Polymeric particulates to improve oral bioavailability of peptide drugs. Molecules.

[21] Brannon-Peppas L (1995). Recent advances on the use of biodegradable microparticles and nanoparticles in controlled drug delivery. Int J Pharm.

[22] Owens DE, Peppas NA (2005). Opsonization, biodistribution, and pharmacokinetics of polymeric nanoparticles. Int J Pharm.

[23] Du Toit LC, Pillay V, Choonara YE, Iyuke SE (2008). Formulation and evaluation of a salted-out isoniazid-loaded nanosystem. AAPS Pharm SciTech.

[24] Kataoka K, Harada A, Nagasaki Y (2001). Block copolymer micelles for drug delivery: Design, characterization and biological significance. Adv Drug Deliv Rev.

[25] Croy SR, Kwon GS (2006). Polymeric Micelles for drug delivery. Curr Pharm Design.

[26] Moghimi SM, Muir IS, Illum L, Davis SS, Kolb-Bachofen V (1993). Coating particles with a block co-polymer (poloxamine-908) suppresses opsonisation but permits the activity of dysopsonins in the serum. Biochim Biophys Acta.

[27] Chen L, Xie Z, Hu J, Chen X, Jing X (2007). Enantiomeric PLA-PEG block copolymers and their stereocomplex micelles used as rifampin delivery. J Nanoparticle Res.

[28] Emanuele AD, Attwood D (2005). Dendrimer-drug interactions. Adv Drug Del Rev.

[29] Duncan R, Izzo L (2005). Dendrimers biocompatibility and toxicity. Adv Drug Del Rev.

[30] Kumar PV, Agashe H, Dutta T, Jain NK (2007). PEgylated dendritic architecture for development of a prolonged drug delivery system for an antitubercular drug. Current Drug Delivery.

[31] Loftsson T, Duchene D (2007). Cyclodextrins and their pharmaceutical applications. Int J Pharm.

[32] Brewster ME, Loftsson T (2007). Cyclodextrins as pharmaceutical solubilizers. Adv Drug Deliv Rev.

[33] Rao KR, Yadav N, Singh J, Krishnaveni Inclusion complex of anti-tubercular rifampicin with beta-cyclodextrin or 2-hydroxypropyl beta-cyclodextrin and a process for producing the same. European Patent EP1581226.

[34] Gregoriadis G, Wills EJ, Swain CP, Tavill AS (1974). Drug carrier potential of liposomes in cancer chemotherapy. Lancet.

[35] Sham JO, Zhang Y, Finlay WH, Roa WH, Lobenberg R (2004). Formulation and characterization of spray-dried powders containing nanoparticles for aerosol delivery to the lung. Int J Pharm.

[36] Deol P, Khuller GK, Joshi K (1997). Therapeutic efficacies of isoniazid and rifampin encapsulated in lung- specific stealth liposomes against *Mycobacterium tuberculosis* infection induced in mice. Antimicro Agents Chemother.

[37] Azarmi S, Roa WH, Lobenberg R (2008). Targeted delivery of nanoparticles for the treatment of lung diseases. Adv Drug Del Rev.

[38] Muttil P, Wang C, Hickey AJ (2009). Inhaled Drug Delivery for Tuberculosis Therapy. Pharmaceutical research.

[39] Visscher GE, Robison RL, Maulding HV, Fong JW, Pearson JE, Argentieri GI (1986). Biodegradation of and tissue reaction to microcapsules. J Biomed Mater Res.

[40] Tice TR, Cowsar DR (1984). Biodegradable controlled-release parenteral systems. J Pharm Technol.

[41] Wu Y, Li M, Gao H (2009). Polymeric micelle composed of PLA and chitosan as a drug carrier. J Polym Res.

[42] Comstock GW (1995). The age of selection of mortality from tuberculosis in successive decades. Am J Epidemiol.

